# Characterisation of Peste Des Petits Ruminants Disease in Pastoralist Flocks in Ngorongoro District of Northern Tanzania and Bluetongue Virus Co-Infection

**DOI:** 10.3390/v12040389

**Published:** 2020-03-31

**Authors:** Bryony Anne Jones, Mana Mahapatra, Chobi Chubwa, Brian Clarke, Carrie Batten, Hayley Hicks, Mark Henstock, Julius Keyyu, Richard Kock, Satya Parida

**Affiliations:** 1 Royal Veterinary College, University of London, Hawkshead Campus, North Mymms, Hatfield AL9 7TA, UK; rkock@rvc.ac.uk; 2 The Pirbright Institute, Ash Road, Pirbright, Woking GU24 0NF, UK; mana.mahapatra@pirbright.ac.uk (M.M.); bdclarke@protonmail.com (B.C.); carrie.batten@pirbright.ac.uk (C.B.); hayley.hicks@pirbright.ac.uk (H.H.); mark.henstock@pirbright.ac.uk (M.H.); satya.parida@pirbright.ac.uk (S.P.); 3 Ngorongoro District Council, Loliondo, PO Box 1 Arusha, Tanzania; chobyclement@hotmail.com; 4 Tanzania Wildlife Research Institute, Box 661 Arusha, Tanzania; julius.keyyu@tawiri.or.tz

**Keywords:** PPR, surveillance, outbreak investigation, differential diagnosis, sheep, goats, ethno-veterinary knowledge

## Abstract

Peste des petits ruminants (PPR) disease was first confirmed in Tanzania in 2008 in sheep and goats in Ngorongoro District, northern Tanzania, and is now endemic in this area. This study aimed to characterise PPR disease in pastoralist small ruminant flocks in Ngorongoro District. During June 2015, 33 PPR-like disease reports were investigated in different parts of the district, using semi-structured interviews, clinical examinations, PPR virus rapid detection test (PPRV-RDT), and laboratory analysis. Ten flocks were confirmed as PPRV infected by PPRV-RDT and/or real-time reverse transcription-polymerase chain reaction (RT-qPCR), and two flocks were co-infected with bluetongue virus (BTV), confirmed by RT-qPCR. Phylogenetic analysis of six partial N gene sequences showed that the PPR viruses clustered with recent lineage III Tanzanian viruses, and grouped with Ugandan, Kenyan and Democratic Republic of Congo isolates. No PPR-like disease was reported in wildlife. There was considerable variation in clinical syndromes between flocks: some showed a full range of PPR signs, while others were predominantly respiratory, diarrhoea, or oro-nasal syndromes, which were associated with different local disease names (*olodua*—a term for rinderpest, *olkipiei*—lung disease, *oloirobi*—fever, *enkorotik*—diarrhoea). BTV co-infection was associated with severe oro-nasal lesions. This clinical variability makes the field diagnosis of PPR challenging, highlighting the importance of access to pen-side antigen tests and multiplex assays to support improved surveillance and targeting of control activities for PPR eradication.

## 1. Introduction

Peste des petits ruminants virus (PPRV, *small ruminant morbillivirus*) causes a highly contagious disease of domestic sheep and goats and some free-ranging and captive wild artiodactyls. Peste des petits ruminants (PPR) is a transboundary disease that is endemic in many parts of Africa and Asia. In the past decade, PPRV has expanded its range into northern, eastern and southern Africa, Central and east Asia [1,2,3,4,5], and most recently to Bulgaria in eastern Europe [6]. PPR is a major threat for small ruminant farmers, making a significant impact on food security, livelihoods and trade [7], and has recently become a target for global eradication by the international animal health community [8].

Among domestic animals, the most important species to be clinically affected by PPR disease are sheep and goats [1], although sporadic outbreaks of PPR disease have been reported in camels [9,10]. Cattle are susceptible to infection but do not show clinical disease, while pigs have been shown to develop clinical disease and transmit virus under experimental conditions [11,12]. It is frequently reported that goats develop more severe disease than sheep [13], although some outbreaks affect both species to a similar degree, or sheep are more severely affected than goats [14,15]. In goats, breed variation in the severity of disease has been demonstrated [16,17]. In wild artiodactyls in Asia, there have been various reports of confirmed PPR disease under natural conditions [18,19] and in zoological collections [20,21], but rarely in Africa [22,23]. However, the role that wildlife play in the epidemiology of PPR is unclear, whether they are infected by virus spillover from domestic animals or whether they can act as bridge or maintenance hosts and contribute to PPRV spread and/or maintenance [24].

The main route of PPRV transmission is through the inhalation of aerosols from sneezing and coughing sick animals, and the incubation period varies from two to six days [14,25]. The virus is excreted by sick sheep and goats from one to two days prior to the development of clinical signs up to at least 10 days after the onset of signs in ocular, nasal and oral secretions at varying levels, and can be detected in faeces after the onset of signs for at least 10 days [14,26,27]. It does not survive long in the environment so indirect transmission plays a minor role [25], and there is no evidence of a carrier state [14]. It is hypothesised that PPRV in the respiratory tract may be taken up by immune cells in the respiratory mucosa, which may migrate to local lymph nodes where viral replication occurs before virus enters the circulation, followed by secondary replication in respiratory and gastro-intestinal epithelial cells [28]. PPRV causes destruction of leucocytes and profound immunosuppression, which leads to opportunistic secondary infections that exacerbate disease severity [29]. Animals that survive PPR infection develop antibodies from 7 to 10 days post-infection. Recovered and vaccinated animals develop lifelong immunity that is fully protective against re-infection [30], and offspring of immune animals have protective maternal antibody for the first three months after birth [13,31].

The clinical signs of PPR can vary from per-acute to sub-acute. The typical acute presentation starts with pyrexia (40–41 °C), lacrimation, serous nasal discharge, depression and anorexia. Diarrhoea starts after 2 to 3 days, causing severe dehydration. Erosions appear on the nasal and oral mucus membranes, with salivation and scabs on the lips. The lacrimation and nasal discharge become profuse and catarrhal, crusting on the eyelids and around the nostrils, sticking the eyelids together and obstructing breathing. There is dyspnoea and coughing due to bronchopneumonia. Pregnant females can abort, and milk yield is reduced [1,17,32]. Death occurs from 10 to 20 days after onset [14,17], and recovery can take several weeks for animals that survive [32]. Morbidity and mortality rates can be very high in naïve populations, with morbidity of up to 100% [33] and case fatality up to 90% [17,25], but these can be much lower in endemic areas or where vaccination is practised, depending on previous exposure, vaccination coverage, innate resistance, body condition, age, animal density, virus strain virulence and secondary bacterial and parasitic infections [14,25].

Due to the diversity of PPR clinical signs, often complicated by secondary infections, there is a long list of differential diagnoses including pasteurellosis, contagious caprine pleuropneumonia (CCPP, caused by *Mycoplasma capricolum* subsp. *capripneumoniae*), bluetongue, orf (contagious pustular dermatitis), sheep and goat pox, foot-and-mouth disease (FMD), other respiratory and enteric bacterial infections, gastro-intestinal parasites, and plant or mineral poisoning [1,17].

Bluetongue virus (BTV) is an *Orbivirus* that is transmitted by *Culicoides* sp. to infect sheep, goats and cattle, and mainly occurs in tropical and sub-tropical regions. In tropical regions, it is generally endemic due to continuous vector transmission, and infection is usually subclinical except in immunologically naive animals that are introduced to the area [34]. Sheep are most susceptible to clinical disease, showing signs that vary from inapparent to severe depending on the virus strain (there are 29 known serotypes [35]) and breed of sheep. For example, indigenous African sheep breeds are generally subclinically infected, but European breeds are more susceptible to disease [34,36].

Animals are infected with BTV by the cutaneous inoculation of virus during the feeding of *Culicoides* sp. The virus replicates in lymph nodes, which is followed by viraemia for up to 20 days, fever and leukopaenia, and then localisation of virus in the vascular endothelium causing hyperaemia, erosions, ulceration and oedema of the lips, buccal mucosa and tongue, gastro-intestinal tract and skin. There may be stiffness and lameness with hyperaemia of the coronary band, cyanosis of the tongue, excess frothy salivation, serous to mucopurulent nasal discharge, and tachypnoea. Goats and cattle are frequently infected but clinical disease is uncommon and mild compared with the signs in sheep [34,36].

In Tanzania, peste des petits ruminants (PPR) disease was first confirmed in 2008 in sheep and goats in Ngorongoro District in the north of the country [37], although, based on serological evidence from a suspected outbreak in 2004, it is possible that the virus had been present in the area before this time [38]. PPR disease was subsequently detected in the centre [39] and south [37,40] of the country and is now considered to be endemic in many parts of Tanzania [41].

During the course of a research project that aimed to investigate the epidemiology of PPRV at the wildlife-livestock interface, in 2014 PPR disease was confirmed in sheep in Ngorongoro Conservation Area (NCA) in the southern part of Ngorongoro District [22]. In the following year, a series of reports of PPR-like disease were received by the Ngorongoro District veterinary services, and so the project collaborated with the district veterinary personnel to investigate some of these reports in order to confirm the presence of PPRV in different parts of Ngorongoro District and to support local and national PPR surveillance. We present here a case series of ten confirmed PPR disease outbreaks—two of which were co-infections with BTV—and describe the variation in clinical syndromes and the disease names used by Maasai livestock keepers in Ngorongoro District. The Maa language disease terminology will be of relevance to veterinary personnel in Maa-speaking areas in northern Tanzania and southern Kenya, and the range of clinical syndromes associated with confirmed PPRV infection will be relevant for clinical surveillance for PPR disease in sub-Saharan Africa, and other areas with extensive small ruminant production systems. 

## 2. Materials and Methods

### 2.1. Study Area

**Figure 1:**
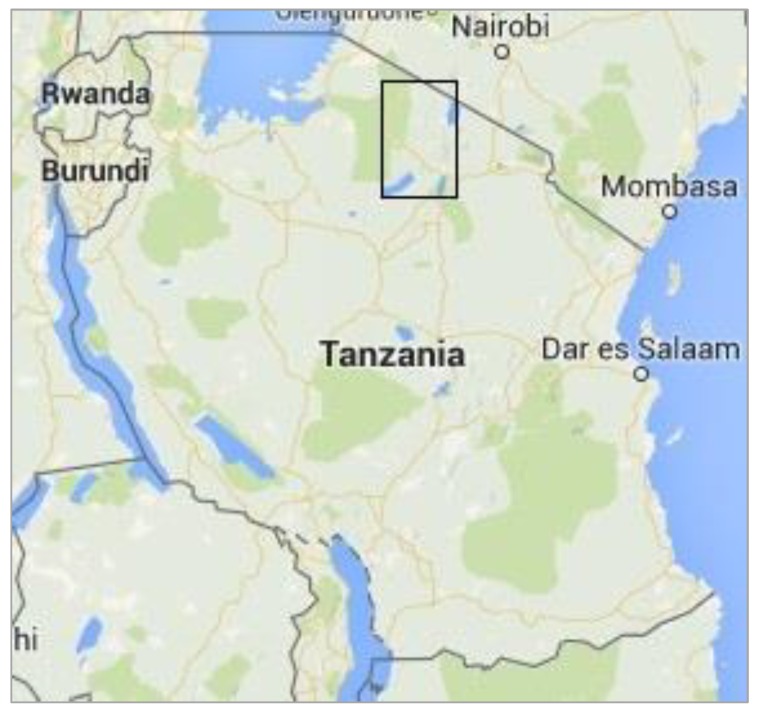
Map of Tanzania. Source: Map data © 2020 Google. The black rectangle indicates the study area in Ngorongoro District in Tanzania, which is shown in more detail in Figure 2.

**Figure 2:**
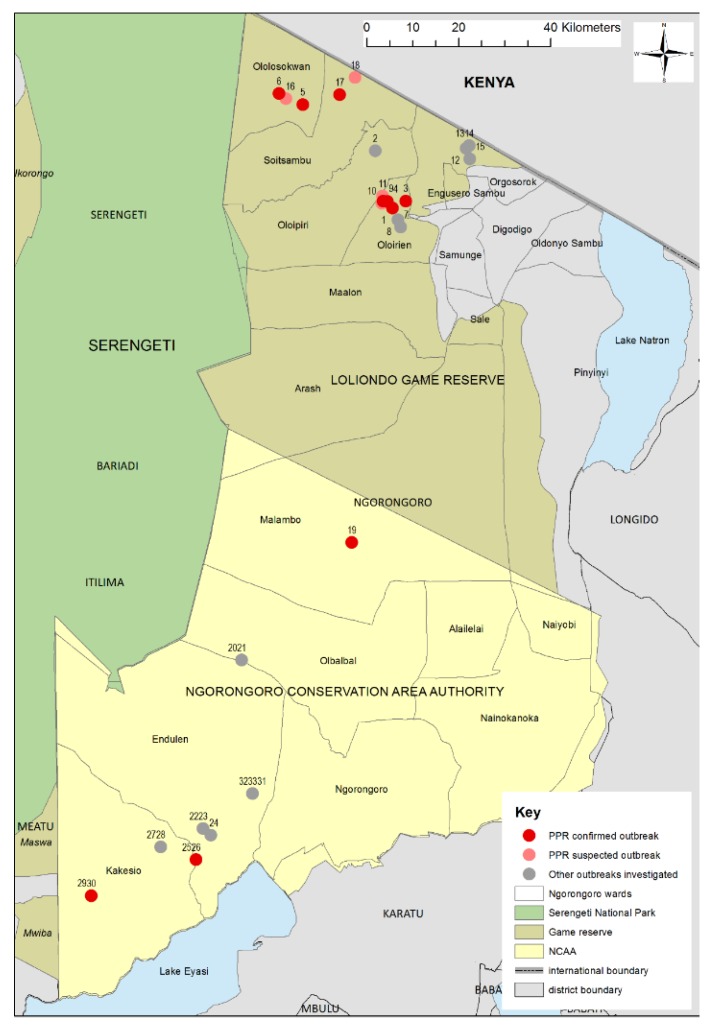
Map of Ngorongoro District showing the location of the flocks where outbreak investigations were carried out. Outbreak locations are represented by coloured circles and the flock number; red = PPRV disease confirmed; pink = PPRV disease suspected due to proximity to confirmed outbreak and similar clinical signs; grey = other outbreaks investigated but not confirmed as PPR. NCAA = Ngorongoro Conservation Area Authority.

Data and sample collection were carried out over a three-week period from June to July 2015 in Ngorongoro District, which is in the Arusha Region of northern Tanzania and is mainly populated by Maasai pastoralist and agro-pastoralists. Ngorongoro is bordered to the north by Narok County of Kenya, to the east and south by Longido, Monduli and Karatu Districts, and to the west by Serengeti National Park and Meatu District (Figure 1 and Figure 2). Ngorongoro Conservation Area Authority (NCAA) covers the southern part of the district, which is a multiple land use area supporting pastoralism, conservation of natural resources and tourism. The north of the district is divided into Loliondo and Sale Divisions where pastoralism and agro-pastoralism is practised. Loliondo Game Controlled Area covers most of these two Divisions. The Serengeti plains extend into the western part of the district and are grazed by resident and migratory wild herbivores and the domestic herds of cattle, sheep and goats of the Maasai pastoralists. The main wildlife species are wildebeest (*Connochaetes taurinus*), zebra (*Equus quagga*), Thomson’s and Grants gazelle (*Eudorcas thomsonii, Gazella grantii*), impala (*Aepyceros melampus*), kongoni (*Alcelaphus buselaphus*), topi (*Damaliscus lunatus*), African buffalo (*Syncerus caffer*) and giraffe (*Camelopardalis giraffe*) [42]. The total population of antelope and buffalo was estimated to be 500,000 based on the Tanzania Wildlife Research Institute (TAWIRI) aerial census in 2010. The small ruminant population in the district was estimated to be 1.25 million in the 2007–2008 agriculture census—655,000 goats and 600,000 sheep [43].

In order to control PPR disease, widespread vaccination against PPRV was carried out in 2011–2012 in northern Tanzania. In Ngorongoro District, it was estimated by the District Veterinary Officer (DVO, third author), based on records of the vaccination campaign, that approximately 70% of the small ruminant population were vaccinated, which led to a reduction in outbreaks. However, the number of cases started to increase again in 2014 and there were many reports of PPR-like disease in the north of the district during the first few months of 2015. In NCAA, where PPR vaccination continued in 2013 and 2014, there were sporadic reports of PPR-like disease. No PPR-like disease had been reported in any wildlife species in any part of the district to the DVO or NCAA.

### 2.2. Outbreak Investigation, PPRV Rapid Diagnostic Test, Sample Collection and Processing

Flocks were selected for outbreak investigation from outbreak reports received shortly before and during the study period by the DVO in Loliondo and by the NCAA veterinary department in NCAA. Thirty-three disease reports were investigated that indicated PPR-like disease, such as reports of mortality of sheep and goats, ocular and nasal discharge, mouth lesions, respiratory signs and/or diarrhoea. Flocks were visited at the household or in the grazing area depending on the time of day, and flock size was estimated by direct observation. For each investigation, a semi-structured interview was conducted with the person or people managing the flock. This covered some or all of the following topics: local Maa language name of disease, date of disease onset, number of animals affected and dead, clinical and post-mortem signs, history of disease in the area, other flocks affected, treatment given, vaccination history, flock contacts and movement, animals brought into the flock in the previous month, and wildlife contact and disease (File S1).

A general examination of the flock was made by observing from a distance and walking among the flock to obtain a general overview of the main clinical signs present in the flock. A selection of sick animals that represented the range of signs observed in the flock overall were clinically examined and their age determined by dentition (number of pairs of permanent incisors present) and owner information. The rectal temperature was recorded, and notes made on the presence and qualitative description of ocular, nasal, oral and respiratory signs, and diarrhoea. 

For animals showing PPR-like signs that had started within the previous 1–3 days, a PPRV rapid detection test (Peste-Test, BDSL Irvine Ltd., UK, [44]) was carried out on conjunctival swabs collected from 1 to 5 animals per flock, depending on the number of suitable animals available. The aim was to obtain at least one positive test result in order to confirm that the disease outbreak in the flock was caused by PPRV. The remaining sample fluid from each test was retained for laboratory analysis.

Additional samples were collected for laboratory analysis from the same animals and a few other sick ones, to give 3–5 animals sampled per flock. Samples collected were: clotted blood for serum, whole blood in EDTA (ethylene diamine tetraacetic acid), conjunctival, nasal and oral swabs (put into virus transport media or phosphate-buffered saline), tissue scrapings from peri-oral skin lesions, and faeces. In some flocks where animals had been sick for several weeks or were recovering, no early cases were found, and so only serum samples were collected from animals that were reported to be recovering from disease. 

In three flocks, a moribund animal was slaughtered for post-mortem examination and tissues samples were collected. 

In total, clotted blood samples were collected from 54 sheep and goats for serological analysis. Conjunctival, nasal and oral swabs (*n* = 31), buffy coats (*n* = 5), PPRV rapid diagnostic test fluid (*n* = 9), faeces (*n* = 11), tissue from peri-oral skin lesions (*n* = 6) and tissue from post-mortem examinations (*n* = 3) were collected from 45 sheep and goats for molecular analysis.

Two flocks (1 and 3) that were confirmed as infected with PPR virus by PPRV rapid detection test (PPRV-RDT) were re-visited one week later to observe the progression of the outbreak; all animals in these two flocks were individually examined, and age (based on dentition), sex, species, and clinical signs were recorded. Unfortunately, due to time constraints, full flock examinations were not carried out on any other flocks that were investigated. Descriptive analysis of the prevalence of clinical signs was carried out and the χ^2^ test was used to explore differences in the prevalence of signs between species and age groups within flocks. Multivariable logistic regression was used to explore the association between presence of clinical signs and species, age and flock. Analysis was carried out in STATA IC version 12.1 (StataCorp LLC). 

All samples were labelled with the date of collection and a unique identification number, placed immediately into a cool box with ice packs, and transported to the base location. The blood samples were processed later the same day: the buffy coats from the EDTA blood samples were collected, and serum was separated from clotted blood. All samples were stored at −20 °C until the end of the fieldwork, after which they were transported to the Tanzania Wildlife Research Institute (TAWIRI) laboratory in Arusha to be stored at −80 °C until shipment in dry ice to The Pirbright Institute, UK. Faeces samples were shipped in dry ice to Centre de Coopération Internationale en Recherche Agronomique pour le Développement (CIRAD), France, where they were stored at −80 °C until analysed. The laboratory analysis of the faecal samples has been reported elsewhere [45].

### 2.3. Antibody Detection by cELISA

The 54 serum samples were tested at The Pirbright Institute for the presence of PPRV-specific antibodies using an in-house anti-hemagglutinin (H) PPRV competitive enzyme-linked immunosorbent assay. Samples with a percentage inhibition value >50% were considered positive as per Anderson and McKay [46]. All the tests were carried out in duplicate wells and borderline positive samples were repeated to confirm results. The mean of the two results from each sample was used in subsequent analysis. 

### 2.4. Real-Time Reverse Transcription-Polymerase Chain Reaction (RT-qPCR)

All clinical samples were screened for the presence of PPR virus nucleic acid by real-time reverse transcription-polymerase chain reaction (RT-qPCR). Nucleic acid was extracted from 100 μL of the swab samples (nasal, ocular and oral) and PPRV rapid diagnostic test fluid by an automated procedure using the LSI MagVet^TM^ Universal Isolation Kit (LSI, Lissieu, France) on the KingFisher TM Flex extraction system (ThermoFisher Scientific, Paisely, UK) following the manufacturer’s instructions. The presence of PPRV nucleic acid was determined by PPRV RT-qPCR following the method of Batten et al. [47]. In addition, nucleic acid samples from swabs of mouth lesions (*n* = 2), biopsy tissues from peri-oral skin lesions (*n* = 4) and post-mortem tissues (*n* = 2, lung, mesenteric and bronchial lymph nodes, spleen) from eight animals in which co-infection with BTV and/or capripoxviruses was suspected were also analysed by RT-qPCR for BTV following the method of Hofmann et al. [48], which detects all known BTV serotypes, and for capripoxvirus by qPCR following the method of Bowden et al. [49], which detects all know capripoxviruses. These samples were taken from flocks 1, 2, 6, 9 and 19 in the Olorien-Magaiduru, Soitsambu and Ololosokwan, and Olbalbal wards (Table 1 and Table S1).

### 2.5. Sequencing and Phylogenetic Analysis

Samples found positive for PPRV by RT-qPCR were selected for gel-based reverse transcription-polymerase chain reaction (RT-PCR) and sequencing, with the aim of determining the lineage of the PPRV and its relationship to PPRV previously detected in the area and wider region. The viral RNA was reverse transcribed, and the C-terminus of the N gene was amplified as previously described [50]. The PCR amplicons were purified using the GE Healthcare Illustra GFXPCR purification kit (GE Healthcare, Buckinghamshire, UK) according to the manufacturer’s instructions. PCR products were sequenced using BigDye^®^ Terminator v3.1 Cycle Sequencing Kit (Applied Biosystems, Carlsbad, CA, USA) using the PCR primers. Sequences were assembled and analysed using SeqMan II (DNAStar Lasergene 8.0). 

The partial N gene sequences (255 nucleotides) available in GenBank for east Africa, including Ethiopia, Eritrea, Uganda, Sudan and Kenya, up to December 2019 were retrieved. The identical sequences were removed, leaving a total of 45 partial N gene sequences that were used along with the six sequences generated in this study to construct a neighbourhood-joining phylogenetic tree. Alignments of the N gene sequences were made using the Clustal W program and used for construction of distance matrices using the Kimura 2-parameter nucleotide substitution model [51] as implemented in the programme MEGA 6.0 [52]. A phylogenetic tree was then generated using MEGA 6.0.

### 2.6. Ethical Approval

Ethical approval for this study was obtained from the Royal Veterinary College Ethics and Welfare Committee on 17/2/2015 (URN 2015 1326). In Tanzania, research permission was provided by TAWIRI and the Tanzania Commission for Science and Technology (COSTECH).

## 3. Results

### 3.1. Outbreak Investigations

A total of 33 outbreak reports were investigated in seven of the 22 wards of Ngorongoro District—Olorien-Magaiduru, Soitsambu, Ololosokwan, and Engoserosambu wards in Loliondo Division, and Olbalbal, Endulen and Kakesio wards in NCAA (Figure 2). Samples were collected from 17 outbreaks and tested by PPRV-RDT and/or RT-qPCR—of which, ten flocks were found to be PPRV positive by one or both tests (Table 1). These confirmed outbreaks were in six of the seven wards where investigations were carried out. In addition, samples from three flocks were positive for BTV by RT-qPCR (flocks 1, 2 and 9), including two flocks that were infected with PPRV (flocks 1 and 9). None of the flocks tested for capripoxvirus were positive. 

Some of the non-confirmed outbreaks might also have been caused by PPRV, but either samples were not collected due to time constraints or lack of suitable clinical cases, or samples were collected but were negative, which could have been due to several reasons such as investigation during the later stages of an outbreak so only animals in late disease could be sampled, or there were few animals suitable for sampling, or deterioration of samples during storage and transportation before laboratory testing. Since it is not possible to distinguish between PPR outbreaks that are not confirmed and true negative outbreaks, we describe below only the confirmed PPR outbreaks. A summary of the results of all the outbreak investigations is provided as Supplementary Information (Table S1). The ten confirmed PPR disease outbreaks are described below grouped into four geographical areas. 

#### 3.1.1. Wasso and the Olorien-Magaiduru Ward

Outbreak reports were investigated in eight flocks in Wasso town and nearby villages in Olorien-Magaiduru ward. PPRV infection was confirmed in four flocks (Flocks 1, 3, 4 and 9, Table 1). Two flocks (Flocks 10 and 11) were also suspected to be affected by PPRV because they were neighbours to PPR-confirmed Flocks 4 and 9 and similar clinical signs were reported and observed, but no samples were collected for confirmation. Another nearby flock (Flock 8) had several kids with the typical skin nodules of goat pox, but no samples were collected for confirmation. The four confirmed outbreaks are described in detail below.

##### Flock 1

**Figure 3:**
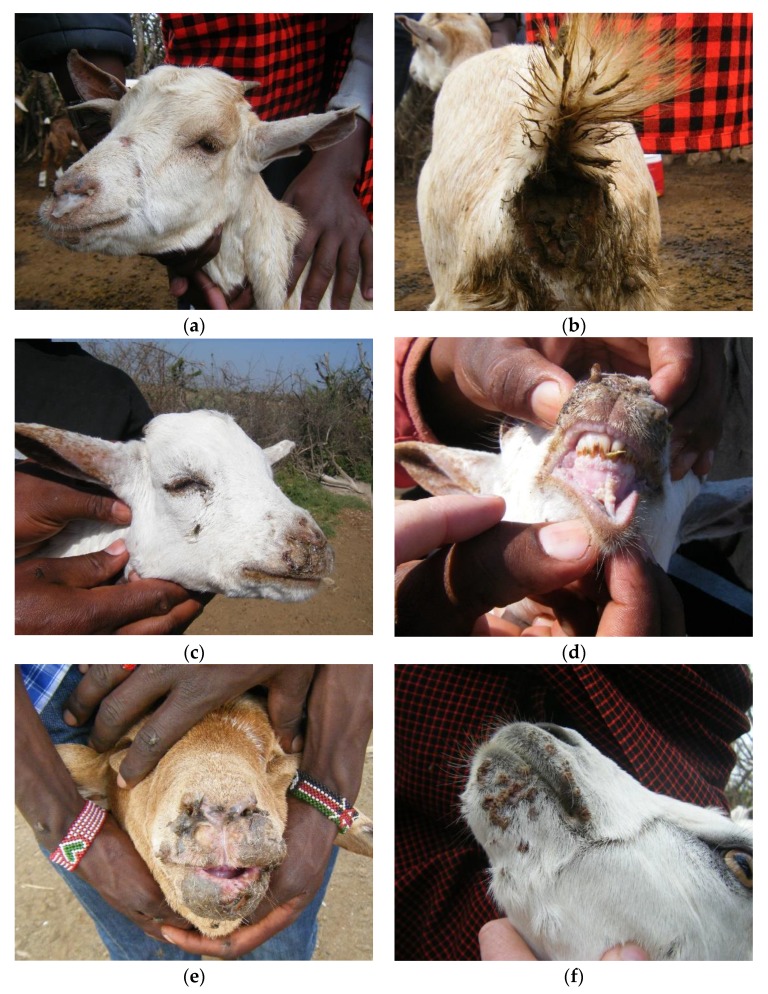
Clinical cases in Flock 1 (confirmed PPRV and bluetongue virus (BTV) infected) in Olorien-Magaiduru ward, showing a range of clinical signs: (**a**) 1–2-year-old goat with mucoid nasal discharge and (**b**) diarrhoea—peste des petits ruminants virus (PPRV) rapid diagnostic test (RDT) and PPRV real-time reverse transcription polymerase chain reaction (RT-qPCR) positive; (**c**) 2–3-month-old goat with lacrimation, nasal discharge, peri-oral skin lesions and (**d**) mouth lesions—not tested; (**e**) 8-month-old sheep with nasal discharge, swollen and ulcerated muzzle and ulceration inside lips and tongue—PPRV-RDT and PPRV RT-qPCR negative; (**f**) 1-year-old goat with peri-oral skin lesions—not tested.

Flock 1 was an unvaccinated flock of 766 animals (479 sheep, 287 goats). The outbreak had started one month earlier, with reported signs of nasal discharge and diarrhoea. The livestock keepers called the disease *olodua,* which means bile or gall bladder in Maa and was the name used for rinderpest in cattle (caused by the related *Rinderpest morbillivirus*, which has been eradicated). General examination of the flock found sheep and goats of all ages with serous or mucoid nasal discharge, sneezing, coughing, and/or diarrhoea (Figure 3a–f).

**Figure 4:**
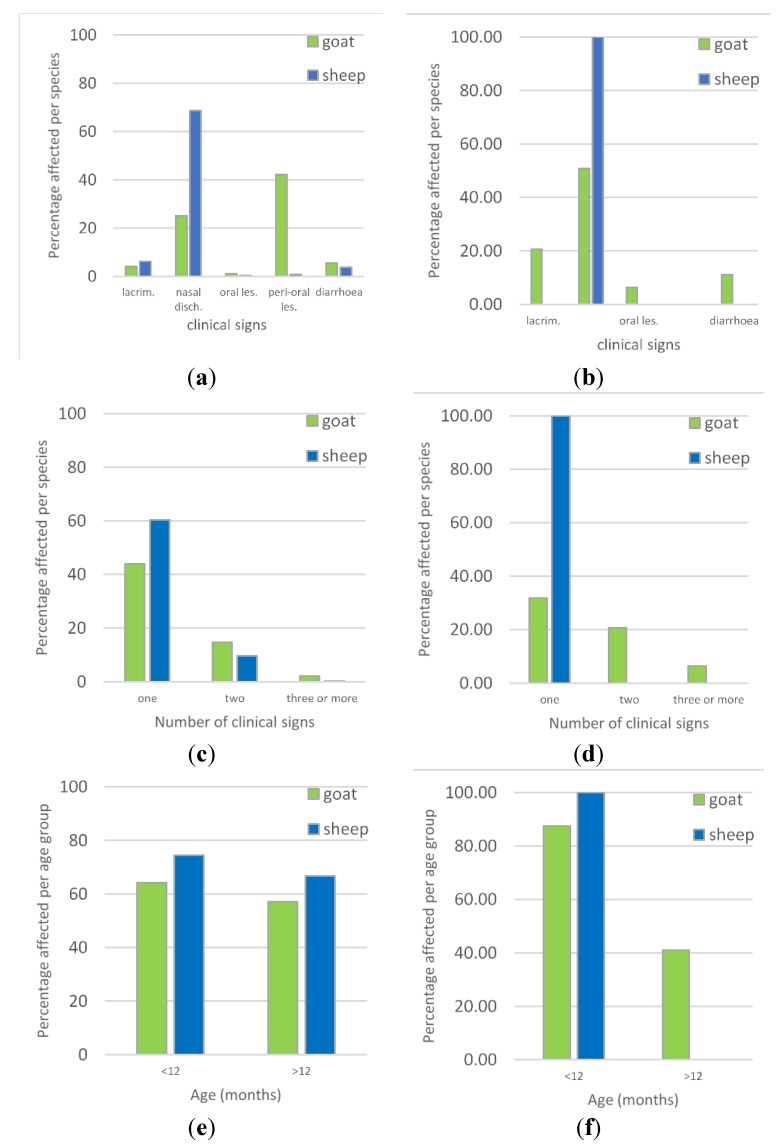
Prevalence of clinical signs in Flocks 1 and 3. (**a**) Prevalence of type of clinical sign in each species in Flock 1 and (**b**) Flock 3; lacrim. = lacrimation, nasal disch. = nasal discharge, oral les. = oral lesions, and peri-oral les. = peri-oral lesions. (**c**) Number of clinical signs per animal by species in Flock 1 and (**d**) Flock 3. (**e**) Prevalence of clinical disease in each age group by species in Flock 1 and (**f**) Flock 3.

Based on the individual examination of all 766 animals in Flock 1, 66.6% of the flock showed clinical signs with a higher proportion of sheep affected (70.2%) than goats (60.6%, *χ*^2^ test p-value = 0.007). The most common sign was nasal discharge with 52.4% of the flock affected, ranging from a slight watery discharge to profuse mucoid or mucopurulent discharge (Figure 3a), which was more frequent in sheep (68.7%) than goats (25.1%, *p* < 0.001) (Figure 4a). A smaller proportion of animals had lacrimation (5.5%), and 4.4% had diarrhoea, based on observed diarrhoea or faecal soiling of the hindquarters (Figure 3b). Coughing and sneezing was commonly heard when moving through the flock, but only one sheep was observed to have dyspnoea. Only a few animals had lesions inside the mouth (0.7%), but small lesions could have been missed (Figure 3d,e). Skin lesions around the mouth and nose (peri-oral lesions) were common in all ages of goats (42.2%) and were highly variable in appearance—large or small nodules, or small ulcers covered with scabs (Figure 3c,f). Very few sheep had peri-oral lesions—only four immature animals were affected (0.8%), and these had swollen muzzles with thickened, ulcerated skin around the nose and mouth, and there was ulceration inside the mouth in two of these cases (Figure 3e).

More than half of the flock (54.1%) had only one clinical sign, which was most commonly nasal discharge (40.5%), followed by peri-oral skin lesions (10.8%), while 11.5% of animals had two or more signs, most commonly nasal discharge combined with peri-oral skin lesions (4.4%), diarrhoea (2.7%) or lacrimation (3.5%).

Although a smaller proportion of goats were affected, they were more likely to have more than one clinical sign (16.7%) compared with sheep (9.8%, *p* < 0.001) (Figure 4c).

There was no difference in the proportions of young (<12 months old, no permanent incisors) compared to adults (>12 months old, one or more pairs of permanent incisors) affected for either goats or sheep (Figure 4e), but among those affected, young animals were more likely to show more than one clinical sign (25.7%) than adults (11.7%, *p* < 0.001) and this applied to both goats (*p* = 0.034) and sheep (*p* = 0.01).

The flock mortality rate was 2.2% during the five-week period from the start of the outbreak to the time of the second visit, and there had been one abortion. The livestock keepers estimated that there had been a 25% drop in milk yield since the start of the outbreak.

Two out of three animals tested were positive by PPRV-RDT, and these were confirmed by PPRV RT-qPCR on rapid test sample fluid and buffy coat. One of two samples from peri-oral scabs in goats was PPRV RT-qPCR positive, and one sample from a sheep that showed similar clinical signs to Figure 3e was both PPRV and BTV RT-qPCR positive, confirming the existence of co-infection of PPRV and BTV. All peri-oral samples were negative for capripoxviruses.

One of the PPRV-RDT-positive animals, a 1-month-old goat, was sacrificed for post-mortem and gross pathology showed two small areas of congested lung and enlarged oedematous mesenteric lymph nodes. No other abnormalities were found.

##### Flock 3

Flock 3 was a smaller flock of 63 goats and 2 sheep that had been vaccinated against PPR two years earlier. The disease problem had started 4 days earlier. The livestock keeper called the disease *olkipiei*, a term that means “lung” because the main sign he had noticed was coughing. *Olkipiei* is usually thought be pneumonia, particularly CCPP, by veterinarians working in the area [53]. However, the livestock keeper had also seen signs of lacrimation and blindness that he did not associate with *olkipiei* and so he was unsure.

**Figure 5:**
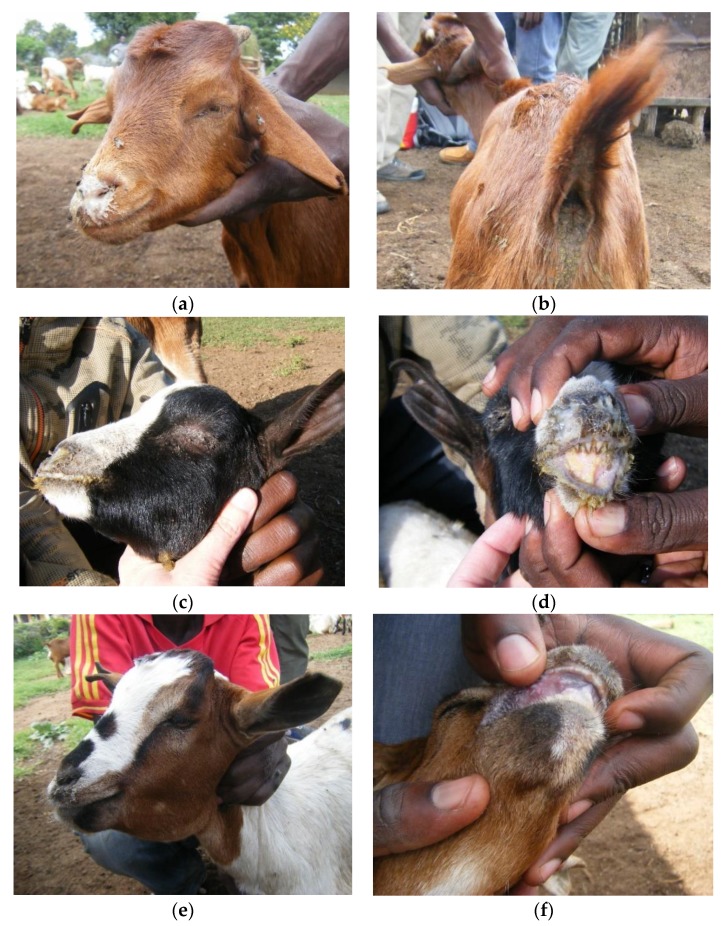
PPR cases in Flock 3 (confirmed PPRV infected) in Olorien-Magaiduru showing a range of clinical signs. (**a**) A 2-year-old goat (G10) with pyrexia, ocular discharge, mucoid nasal discharge blocking nostrils, and (**b**) diarrhoea and straining—peste des petits ruminants virus (PPRV) rapid diagnostic test (RDT) and PPRV real-time reverse transcription polymerase chain reaction (RT-qPCR) positive; (**c**) 3-month-old goat with ocular discharge (eyelids stuck together), nasal discharge, and (**d**) mouth lesions—not tested; (**e**) 6-month-old goat (G11) with pyrexia, sub-mandibular oedema, lacrimation, nasal discharge and (**f**) ulcers on lip margin and necrotic lesion upper gum—PPRV-RDT and PPRV RT-qPCR positive.

The main signs observed during individual examination of all animals were lacrimation, nasal discharge, coughing, sores in the mouth and around the muzzle, sub-mandibular swelling and diarrhoea (Figure 5). As in Flock 1, there was high morbidity, with 60% of animals affected, and low mortality (1.5% during the two-week period from the start of the outbreak to the time of the second visit). However, a higher proportion of goats had multiple signs (26%) compared to Flock 1 (Figure 4d). Nasal discharge was the most common sign, with 52% of the flock affected, but lacrimation (20%) and diarrhoea (11%) were more common than in Flock 1 (Figure 4b). A few animals (4) had mouth or lip lesions and two had sub-mandibular oedema. In sharp contrast to Flock 1, no animals had peri-oral skin lesions. Unlike in Flock 1, young goats (<12 months) were more likely to be affected (87.5%) than adults (41.0%, *p* <0.001) (Figure 4f), but there was no evidence of a difference in the number of clinical signs per animal between young and adults among affected animals.

Two out of three animals tested by PPRV-RDT were positive, and these were confirmed by PPRV RT-qPCR of rapid test sample fluid, swabs and/or buffy coat. The PPRV-RDT-negative animal was later found to be PPRV positive when the rapid test fluid was tested by PPRV RT-qPCR, demonstrating the lower sensitivity of PPRV-RDT compared with RT-qPCR.

Combining the results of flock examinations of Flocks 1 and 3, univariable analysis found no evidence of a difference in prevalence of clinical signs between the two flocks, but sheep were more likely to be affected than goats, and young (<12 months) were more likely to be affected than adults, (>12 months) (Table 2). A multivariable logistic regression model with flock as fixed effect, showed that sheep were more likely to have clinical signs than goats when adjusted for age (adjusted odds ration (OR) 1.60, 95% CI 1.17, 2.17, *p* = 0.003) and young animals were more likely to have clinical signs than adults when adjusted for species (OR 1.61, 95% CI 1.20, 2.16, *p* = 0.001).

##### Flock 4

Flocks 4, 9, 10 and 11 were located near to each other. Flock 4 was a mainly goat flock of approximately 200 animals, that was last vaccinated in 2012. The livestock keeper thought the disease was a mild form of *olodua*, with reported signs of emaciation, diarrhoea and lesions around the mouth in a small number of animals (5.5% of the flock) and 3% flock mortality. Only three cases were examined—one with nasal discharge and diarrhoea, one with diarrhoea and emaciation, and one with diarrhoea and peri-oral skin lesions, which was PPRV-RDT positive.

##### Flock 9

Flock 9 was a large flock of approximately 400 sheep and 250 goats that was vaccinated in 2012 and had been vaccinated privately one month earlier together with the nearby Flock 10, although the details of the vaccination were unclear. The flock was affected by a disease that the livestock keeper called *oloirobi*, which means “fever” and is a term often associated with foot-and-mouth disease (FMD)-like signs. The main signs reported by the livestock keeper were “wounds” in the mouth, diarrhoea, throat swelling and bloat, but on flock examination, many sheep and goats were also observed to have sneezing, coughing and nasal discharge. The signs were apparently more severe in sheep than goats, with higher morbidity (20%) and mortality (15%) reported in young sheep with oral lesions and diarrhoea, whereas goats showed mainly upper respiratory signs and no reported mortality.

**Figure 6:**
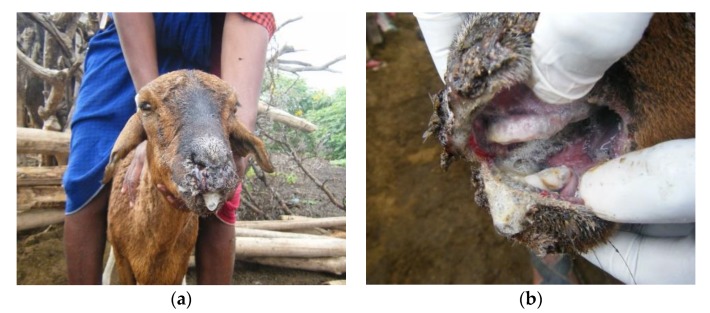
(**a**) A 4-year-old sheep with nasal discharge, frothy salivation, swollen and ulcerated muzzle; (**b**) extensive ulceration of lips and buccal cavity, and diarrhoea. This sheep was peste des petits ruminants virus (PPRV) real-time reverse transcription polymerase chain reaction (RT-qPCR) negative and bluetongue virus (BTV) RT-qPCR positive. It was in Flock 9 (confirmed PPRV and BTV infected) in Olorien-Magaiduru, in which two other sheep were PPRV RT-qPCR positive.

One 4-year-old sheep had profuse mucoid nasal discharge, profuse frothy salivation, extensive sores covering most of the muzzle and lips, a cheesy coating on the dental pad and the floor of the mouth, and watery green diarrhoea (Figure 6). It was found to be PPRV RT-qPCR negative but BTV RT-qPCR positive. Three animals tested by PPRV-RDT were negative, but two of these were PPRV RT-qPCR positive (rapid test sample fluid from a sheep, swabs from a goat). As for Flock 1, the concurrent detection of both BTV and PPRV by RT-qPCR suggests that this was a mixed infection, which might explain the extensive muzzle and mouth lesions seen in sheep but not goats in this flock.

#### 3.1.2. Soitsambu and Ololosokwan Wards

In Soitsambu and Ololosokwan wards, to the northwest of Wasso and Olorien-Magaiduru, six outbreaks were investigated. PPRV infection was confirmed in three flocks (Flocks 5, 6 and 17) by PPRV-RDT and/or RT-qPCR, and two of the flocks (Flocks 16 and F18) were strongly suspected to be affected by PPR based on proximity to confirmed outbreaks and the similarity of clinical signs. As in Olorien-Magaiduru ward, there was variation in the disease syndromes between flocks with confirmed PPRV, both in the owner description and in observed signs. The disease in the remaining flock (Flock 2) was suspected to be CCPP based on clinical and post-mortem signs.

##### Flock 5

Flock 5 was an unvaccinated flock of approximately 370 sheep and 100 goats. The livestock keeper called the disease *olodua* and said that it was only affecting adult sheep, causing profuse frothy salivation and nasal discharge. Twenty animals were affected (4.3% morbidity) and one had died (0.2% mortality). Clinical examination of three affected immature sheep showed signs of profuse frothy salivation, mucoid nasal discharge, ulceration of the tongue and gums with white coating, and ulceration of the lips and the muzzle. PPRV-RDT was carried out for two of these animals and one was positive, while swabs from both animals were PPRV RT-qPCR positive (but not tested for BTV).

##### Flock 6

Flock 6 numbered approximately 500 sheep and goats and was reported to be last vaccinated in 2012. It belonged to a relative of the owner of Flock 5 which was located approximately 5 km away. The livestock keeper described the disease as affecting sheep and goats of all ages, causing diarrhoea, mouth lesions and dyspnoea. He was unsure whether the disease problem was *olodua* or *olkipiei*, observing that *olkipiei* does not normally affect sheep.

Four clinical cases were examined, which were young sheep and goats (4–10 months old). The two goats had nasal discharge and diarrhoea, and one of these also had peri-oral skin lesions. The two sheep had pyrexia, lacrimation and nasal discharge, and one had severe dyspnoea (very laboured breathing with abdominal lift) and tachypnoea. One of the sheep was PPRV-RDT positive, and swabs from this animal and one of the goats were PPRV RT-qPCR positive.

##### Flock 17

Flock 17 was in a village located approximately 6 km north of flock 5, where PPR vaccination had been carried out in 2012. The disease, for which the owner did not have a name, was affecting sheep more than goats, with sheep reported to have “wounds” in the mouth and blood-stained nasal discharge, while a few goats had diarrhoea. Clinical examinations found both young and adult sheep with nasal discharge, erosion of the dental pad and salivation. One 4-month-old sheep had pyrexia, profuse lacrimation, nasal discharge, and hair loss and dried discharge around the nose and mouth. A 6-month-old goat had lacrimation, peri-oral skin lesions and diarrhoea. These two animals were both PPRV RT-qPCR positive.

##### Flock 2

Flock 2 was in a village to the southeast of Soitsambu. It was affected by a disease that the owner called *olkipiei* mixed with *olodua*, which he said was only affecting adult goats. He reported that they first showed signs of bloat, then diarrhoea, mouth lesions, lacrimation, and respiratory difficulty. Out of a flock of approximately 180 goats and 60 sheep, approximately 60 goats had died. The flock had been vaccinated against PPR two years earlier. At the time of the outbreak investigation, 14 adult goats were observed to be sick with one or more signs of diarrhoea, nasal discharge, pyrexia, lacrimation or dyspnoea. One PPRV-RDT was negative for an adult goat with pyrexia, slight lacrimation, slight watery nasal discharge and tachypnoea (82 respirations per minute). One 3-year-old goat that had severe dyspnoea (laboured breathing with abdominal lift and mouth breathing) was sacrificed for post-mortem examination, and showed classical signs of CCPP; a large yellow fibrin clot in the ventral thorax and almost complete hepatisation of the lung with adhesions to the thoracic wall (Figure S1). Samples of lung tissue were PPRV RT-qPCR negative and capripox qPCR negative but were found to be positive for BTV. The samples were not tested for CCPP.

#### 3.1.3. Olbalbal Ward

Two reports of PPR-like disease were received by NCAA in Olbalbal ward—one from the north in the Gol Mountains (Flock 19) where PPRV infection was confirmed by RT-qPCR, and one from near to Olduvai (Flocks 20 and 21) where PPRV was not confirmed.

##### Flock 19

Flock 19 was a flock of 800 sheep and goats that was last vaccinated in 2013. The livestock keeper called the disease *olkipiei*. It had only affected goats—200 animals were reported to have died during the previous two months and 50 animals were found to be sick during our visit. He reported the main signs to be difficulty in breathing and bloat followed by diarrhoea and death. Approximately 30 goats had aborted since the outbreak started. He had observed congested lungs and an enlarged gall bladder at post-mortem. Clinical cases in four adults (2–5 years old), four immature (8–12 months old) and 31 young (1–3 months old) goats were examined. The adults and immature goats all had nasal discharge, together with pyrexia, dyspnoea, and/or diarrhoea or bloat. In the young kids, nasal and eye discharge, peri-oral skin lesions, and granulomatous and ulcerated lesions in the mouth were the predominant signs, with no diarrhoea or respiratory signs. Two animals were sampled and were found to be negative by PPRV-RDT, swabs were negative in PPRV RT-qPCR, but out of two tissue samples collected from peri-oral skin lesions of young kids, one was PPRV RT-qPCR positive.

#### 3.1.4. Endulen and Kakesio Wards

Twelve flock visits were made in Endulen and Kakesio wards, in the southern part of NCAA, and PPRV infection was confirmed in two flocks (Flocks 26 and 29). Five flocks were visited in Laetoli village in Endulen ward—one of which was confirmed by RT-qPCR to have PPRV infection.

##### Flock 26

Flock 26 contained approximately 300 sheep and goats and was grazing together with Flock 25. Neither flock had been previously vaccinated against PPR. Both sheep and goats were reported to have had diarrhoea during the previous month, and four kids had died. Nine cases in sheep and goats were examined from the two flocks during our visit. Six adult and immature (7-month-old) sheep and one adult goat had two or more signs of pyrexia, dried ocular discharge, congested mucous membranes, nasal discharge, and diarrhoea. One of these sheep had aborted the day before. One adult sheep and one 4-month-old goat appeared to be weak and had erosive lesions on the hard palate and inside the lips, but no other signs. One out of six ocular and nasal swabs was PPRV RT-qPCR positive.

##### Flock 29

Two flocks were visited in Endulele village, Kakesio, and one was confirmed by RT-qPCR to be infected with PPRV. Flock 29 was a large unvaccinated flock of approximately 500 sheep and goats. The disease problem, *enkorotik*, which means diarrhoea, had started approximately one month earlier, affecting both sheep and goats, with high mortality in kids. The main signs reported were watery blackish diarrhoea, with mucoid nasal discharge and mouth lesions. Eight clinical cases were examined. Two adult goats had nasal discharge and one had lacrimation, and an adult sheep had diarrhoea. One 8-month-old sheep, in poor condition with ocular and nasal discharge, showed a weak positive result in PPRV-RDT. One 2-month-old kid had diarrhoea, another had lacrimation, nasal discharge and diarrhoea, and a young lamb had nasal discharge and a lesion on the lower gum. This lamb was PPRV-RDT negative but PPRV RT-qPCR on the rapid test sample fluid was positive.

### 3.2. Overview of Clinical Syndromes and Maa Disease Names

During the study period (June–July 2015), PPRV infection and disease was confirmed in domestic flocks in both the north and south of Ngorongoro District, and co-infection of PPRV and BTV was found in two flocks in the north (Flocks 1 and 9).

The clinical presentation of confirmed outbreaks varied within wards and across the district, and several different local names were used by livestock keepers (Table 1). Some of the confirmed outbreaks in the northern wards showed a range of typical PPR signs among affected sheep and goats—pyrexia, lacrimation, nasal discharge, mouth lesions, coughing, sneezing, pneumonia and diarrhoea (Flocks 1, 3 and 6) and were called either *olodua* (bile/gall bladder) or *olkipiei* (lung). Some signs were only present in a few flocks; dyspnoea (Flocks 5, 6 and 19) and sub-mandibular oedema (Flock 3).

In some flocks, there were severe oro-nasal lesions in sheep in which the buccal mucosa was almost entirely eroded and necrotic with profuse frothy salivation, profuse nasal discharge, swollen ulcerated muzzles and necrotic mouth lesions, and sheep were more frequently affected than goats in most of these flocks (Flocks 1, 5, 9, 17). Two of these flocks had PPRV and BTV co-infections (Flocks 1 and 9, Table 1). These outbreaks were called either *olodua* or *oloirobi* (fever).

By contrast, in one flock (Flock 19, Olbabal), the disease, *olkipiei*, was mainly affecting goats and the main reported signs were respiratory signs with diarrhoea, although, during clinical examination of kids, signs of lacrimation, nasal discharge, and peri-oral and oral lesions were found.

In the southern wards, Endulen and Kakesio, diarrhoea was the main sign reported and the disease was called *enkorotik* (diarrhoea) but, on close examination, a few animals also had nasal discharge, lacrimation or mouth lesions.

Among the ten PPR confirmed flocks, morbidity ranged from 4% to 67%, while flock mortality ranged from 0.2% to 4%—except for one of the flocks that was affected by the severe oro-nasal syndrome and BTV co-infection, which had 9% mortality (Flock 9), and Flock 19 with the respiratory syndrome, which had 25% mortality (Table 1).

### 3.3. Comparison of PPRV-RDT and PPRV RT-qPCR Results

Out of the 17 clinically sick animals that were sampled and tested by PPRV-RDT, 15 were also tested by PPRV RT-qPCR. Of these, six animals were positive in both tests and four animals were negative in both tests, while five animals were negative in PPRV-RDT but positive in PPRV RT-qPCR—one animal in each of Flocks 3, 5 and 29 and two animals in Flock 9 (Table 3). No animals were positive in PPRV-RDT and negative in PPRV RT-qPCR. In relation to PPRV RT-qPCR, which is considered to be highly sensitive and specific [47], for the small number of samples from clinical cases collected during this study, the specificity of PPRV-RDT was 100.0% (95% CI 39.8%, 100.0%) and the sensitivity was 54.5% (95% CI 23.4%, 83.3%), while the positive predictive value was 100% (95% CI 54.1%, 100.0%) and the negative predictive value was 44.4% (95% CI 13.7%, 78.8%). However, these figures are based on a small number of samples (*n* = 15).

### 3.4. Molecular Sequencing

**Figure 7:**
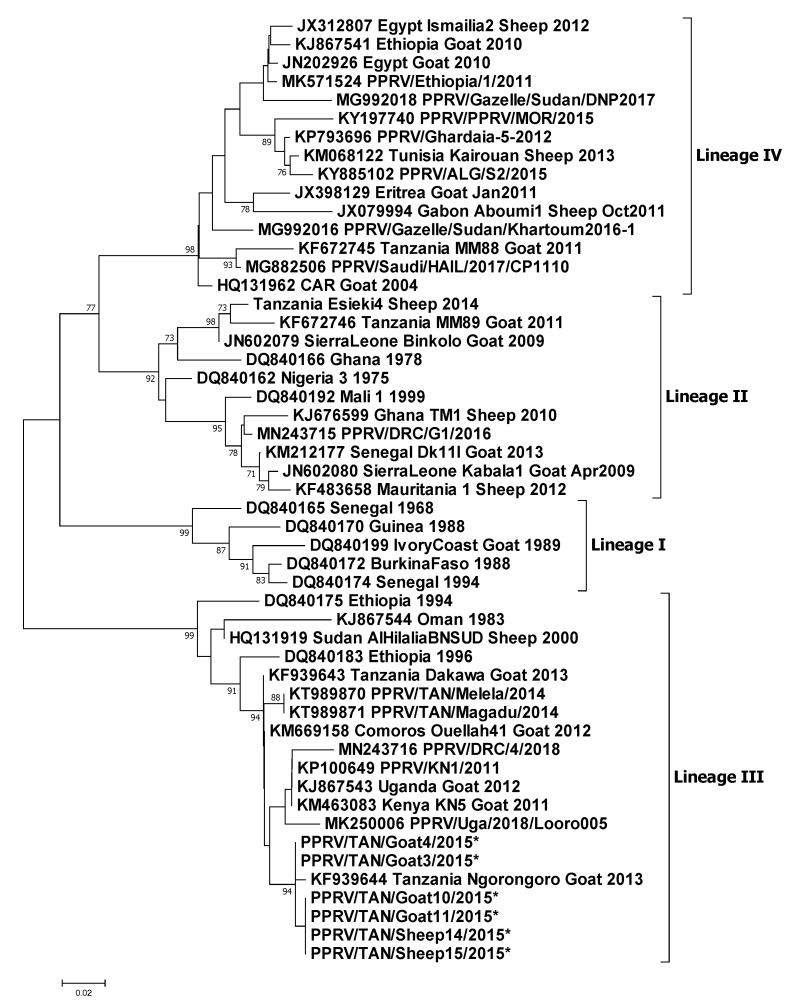
Neighbour-joining tree constructed on the basis of partial N gene sequences of the peste des petits ruminants virus (PPRV). The tree shows the relationships among the African PPRV isolates. The scale bar indicates nucleotide substitutions per site. The Kimura 2-parameter model with percentage of replicate trees in which the associated taxa clustered together in the 1000 bootstrap replicates is shown next to the branches. The six sequences generated in this study (accession numbers: MT181842-47) are indicated by an asterisk at the end of the taxon name. The taxon name of the sequences retrieved from GenBank contains the accession number followed by the name of the country and the year of isolation.

Selected samples that were positive in PPRV RT-qPCR were sequenced on both the strands. A total of six partial N gene sequences were generated in this study—four from goats and two from sheep. These sequences have been deposited in GenBank with accession numbers MT181842-47. The six sequences along with the 45 sequences obtained from GenBank were analysed further to construct a phylogenetic tree that confirmed these isolates to be of lineage III origin (Figure 7). These six sequences were most closely related to the lineage III virus circulating in Ngorongoro District in 2013 (KF939644) and grouped with other east African lineage III viruses circulating in Uganda, Kenya and the Democratic Republic of Congo.

The identical sequences, PPRV/TAN/Goat10/2015 and PPRV/TAN/Goat11/2015, were from two goats from Flock 3 in Wasso (pictured in Figure 3a,b; G10, and Figure 3e; G11), and these were identical to PPRV/TAN/Sheep14/2015 from a sheep in Flock 5 and PPRV/TAN/Sheep15/2015 from a sheep in Flock 6, which were approximately 5 km apart in Ololosokwan ward. These three flocks had different clinical presentations. In Flock 3, which was mainly goats, the main signs were lacrimation, nasal discharge, coughing, sores in the mouth and around the muzzle, sub-mandibular swelling and diarrhoea. In Flock 5, sheep were predominantly affected with profuse frothy salivation, nasal discharge, and severe oral inflammation and ulceration, while Flock 6 showed a range of signs in sheep and goats; lacrimation, nasal discharge, dyspnoea, mouth lesions and peri-oral skin nodules, and diarrhoea.

Two identical sequences, PPRV/TAN/Goat3/2015 and PPRV/TAN/Goat4/2015, differed slightly from the above sequences: these were from Flock 1 (in which BTV was also detected by RT-qPCR), which was located approximately 1 km away from Flock 3, but the outbreak in Flock 1 had started approximately 3 weeks earlier than in Flock 3. There were similar clinical signs in these outbreaks, except for a high prevalence of peri-oral skin lesions in goats of Flock 1 (42.2% of 287 goats, 0.8% of 479 sheep) that were not present in Flock 3 (Figure 4a,b).

## 4. Discussion

We have described a series of ten confirmed PPR disease outbreaks in Ngorongoro District of northern Tanzania that occurred within a short time frame and showed a variety of clinical syndromes. The syndromes included a classic PPR syndrome, with sheep and goats showing a range of signs—fever, ocular and nasal discharge, sneezing and coughing, mouth lesions and diarrhoea, a respiratory syndrome that mainly affected goats, a diarrhoea syndrome of sheep and goats, and a severe oro-nasal syndrome of sheep, with milder signs in goats. The oro-nasal syndrome was associated with PPRV and BTV co-infection, which has not previously been described in east Africa.

These confirmed PPR outbreaks were identified from a non-random selection of a small number of outbreak reports. Therefore, the findings are not necessarily representative for the area and care should be taken when drawing generalised inferences or extrapolating to other populations. However, the detailed descriptions of these outbreaks provide examples of how variable the clinical presentation of PPR disease can be, which is of relevance to researchers and veterinarians and para-veterinarians involved in disease surveillance in other PPR-endemic areas. Another limitation of the investigations was differential diagnosis: a limited number of samples were tested for BTV and capripox, but ideally other possible co-infections would also have been investigated by laboratory diagnostics, such as orf (contagious pustular dermatitis) and CCPP.

Given the relatively recent introduction of PPRV to Tanzania and some suppression of disease occurrence through vaccination, it is not surprising that there is variation in the local Maa language terms for PPR disease. For the confirmed outbreaks described in this study, the variation in names used by the livestock keepers appears to be explained by the variation in clinical syndromes. In this study, out of the ten confirmed outbreaks, three livestock keepers called the disease *olodua* (“bile” or “gall bladder”, a term used for rinderpest in cattle) for outbreaks where there was a range of signs including lacrimation, nasal discharge, mouth lesions, coughing, sneezing and diarrhoea. Two livestock keepers called it *olkipiei* (“lungs”, a term used for pneumonia in several species) for outbreaks where coughing and respiratory signs predominated. One livestock keeper called it *oloirobi* (“fever”, a term associated with foot-and-mouth disease), where sheep were affected with severe oro-nasal syndrome, and one called it *enkorotik* (diarrhoea) where the predominant sign was diarrhoea. Another livestock keeper was not sure whether the disease was *olodua* or *olkipiei* because of the mixture of ocular, nasal, oral, respiratory and diarrhoea signs that he saw. Considering all the 33 outbreak investigations carried out during this study, these Maa disease terms were used consistently for particular syndromes—*enkorotik* for diarrhoea, *olkipiei* for respiratory difficulty, *oloirobi* for lesions in the mouth and *olodua* for rinderpest-like disease in sheep and goats.

These findings are of relevance in the Maa-speaking areas of northern Tanzania and southern Kenya, although there may be local variations, and so veterinary personnel working in the field should engage with livestock keepers to understand the local disease terminology and the characteristics of the syndromes associated with the terms used in the local area.

This variation in clinical signs and the use of local disease names that are usually associated with other small ruminant diseases such as CCPP, FMD and parasitic gastro-enteritis [53,54,55] have led to uncertainty in clinical diagnosis for field veterinarians and other animal health personnel. Outbreaks with a range of typical PPR signs may be correctly diagnosed clinically as PPR, but outbreaks in which respiratory or diarrhoea signs predominate may be mis-diagnosed. Laboratory confirmation is rarely attempted, and so clinical diagnoses are not verified. From the confirmed outbreaks described in this study, it is clear that there is a major overlap in clinical signs between PPR and other important diseases such as bluetongue, FMD, CCPP and other causes of pneumonia, which causes genuine uncertainty for field veterinarians, and highlights the need for improved access to pen-side diagnostic tests and laboratory diagnostics for rapid diagnosis and action. Greater experience of the variation in clinical presentation of confirmed PPR disease in the local context would lead to improvement in the specificity and sensitivity of clinical diagnosis by field veterinary personnel. The overlap between PPR disease and other diseases and the uncertainty of clinical diagnosis of PPR has previously been observed in the pastoralist Afar Region of Ethiopia [56], and is likely to be relevant in other pastoralist areas. In such areas, it will be important to document local disease names associated with PPR-like disease and to confirm the cause of disease with pen-side or laboratory diagnostic tests, to increase the sensitivity and specificity of PPR surveillance, which is important for understanding the incidence and spatial distribution of PPR for targeting of vaccination and monitoring the effect of vaccination as part of the PPR global eradication programme.

The variation in clinical presentation of PPR has implications for the development of a clinical case definition for PPR-like disease for use in PPR passive and active surveillance to identify possible PPR cases for further investigation and confirmation by rapid detection test or laboratory assay. In the study area, the case definition should be very broad to be sufficiently sensitive to capture the majority of PPR cases, but needs to be supported by PPRV rapid detection tests to increase specificity. When disease reports are received from livestock keepers, veterinarians will need to consider reports of respiratory syndromes, diarrhoea syndromes and oro-nasal syndromes as possible PPR cases for further investigation.

The results of this study demonstrate the lower sensitivity of PPRV-RDT when compared to RT-qPCR as observed by Baron et al. [43]. The RT-qPCR targets the nucleocapsid (N) gene of PPRV of all four lineages. It is highly specific and sensitive and shows no cross-reactivity with other morbilliviruses [47]. PPRV-RDT uses a monoclonal antibody C77 that recognises the H protein of PPRV of all four lineages and has a sensitivity of 83.54% (95% confidence interval, CI 73.5, 90.0%) and specificity of 94.59% (95% CI 86.7, 98.5%) relative to RT-PCR/RT-qPCR [44]. Under experimental conditions, PPRV-RDT is able to detect the presence of PPRV antigen as early as 4 days post-infection (dpi) when animals have increased body temperature but no other clinical signs, and at 7 dpi when animals have clinical signs of ocular and nasal congestion and discharge [44]. The lower sensitivity of PPRV-RDT indicates that some PPRV-excreting animals may show a false-negative result in PPRV-RDT but are positive by RT-qPCR. It is also possible for an animal to have a positive PPRV-RDT result but a negative RT-qPCR result due to variation in the ratio of viral antigen to viral genome in different animals, due to factors present in the samples that are inhibitory in RT-qPCR [44], or due to a PPRV-RDT false positive that is a true negative by RT-qPCR.

In four of the flocks that were investigated, one or more animals was PPRV-RDT negative but PPRV RT-qPCR positive. For three of these flocks, one or more of the other animals sampled was positive by PPRV-RDT and therefore the outbreak was confirmed as PPRV infection at the time of the investigation. However, for one flock, three animals were tested by PPRV-RDT and all were negative, but two of these were later found to be positive by PPRV RT-qPCR. Therefore, if an investigation relies solely on PPRV-RDT for confirmation, some PPRV outbreaks might be mis-diagnosed as negative for PPRV. In this case, it is important to test several animals with early clinical signs by PPRV RDT to increase the likelihood of obtaining a positive test if the flock is PPRV infected.

For field veterinarians using PPRV-RDT, it is important that they are trained in how to select animals with early clinical signs for testing and to understand the limitations of the test. It should be used as part of a comprehensive outbreak investigation that includes history taking and clinical examination. Animals that are in the early stages of PPR clinical disease (1–5 days after onset with pyrexia, nasal discharge, and lacrimation), when virus excretion is highest, should be selected for testing to increase the chances of finding an animal that is excreting sufficient quantities of virus to be detected by the test. Due to the low negative predictive value, if the first animal that is tested is negative, then several more should be tested until a positive animal is detected. If no tests are positive, this does not necessarily mean that the flock is not infected with PPRV. If the outbreak investigation suggests a strong suspicion of PPR disease, then samples should be collected for laboratory testing using a more sensitive assay (immunocapture ELISA, RT-PCR or RT-qPCR) before concluding that the flock is likely to be PPRV negative. If an investigation is conducted on an outbreak that has been continuing for a while, it may not be possible to find suitable early cases for PPRV-RDT—in which case, samples should be collected for laboratory diagnosis. In addition, since PPRV is transmitted by direct contact between infected and susceptible flocks, the investigator should visit neighbouring or in-contact flocks to determine whether they are also affected by PPR-like disease and search for early cases for rapid diagnostic testing.

In this study, conjunctival swabs were used in PPRV-RDT. However, a recent experimental infection study that compared the use of conjunctival and nasal swabs for PPRV RT-qPCR demonstrated that nasal swabs were superior for the detection of PPRV nucleic acids from two days post-infection to 14 days post-infection when the study ended [27]. Therefore, nasal samples may be preferable for PPRV-RDT in order to detect the disease during outbreak investigations.

Bluetongue virus was detected by RT-qPCR in two of the PPRV-confirmed flocks, indicating that some of the variation in clinical signs may have been due to co-infection of PPRV and BTV. This area is assumed to be a BTV-endemic area, and so clinical bluetongue disease would only be expected in naïve animals such as those introduced from BTV-free areas [34,36]. However, PPRV is known to have an immunosuppressive effect [32], and so perhaps the co-infection of BTV and PPRV could have led to clinical bluetongue disease? PPRV and BTV co-infection has previously been reported in India [57,58]. In 2016, one year after this study, Kgotlele et al. [59] investigated an outbreak of respiratory disease in Loliondo and using a multiplex assay found co-infections of PPRV with *Mycoplasma capricolum* subsp. *capripneumoniae* (causative agent of CCPP), capripoxvirus and *Pastuerella multocida*. In this study, we did not assay for these pathogens, but sheep and goat pox and CCPP were diagnosed based on typical clinical signs in flocks that neighboured confirmed PPRV-infected flocks. Co-infection of PPRV with capripoxvirus has been reported in the Democratic Republic of Congo [60] and in India [61,62]. Peri-oral skin lesions were present in some flocks but not others, which raises the question of whether the lesions were due to PPRV infection or due to co-infection with other pathogens such as orf virus or bacterial infections. Co-infection of PPRV and orf virus has previously been reported in India [63].

The variation in PPR clinical signs and co-infection described in this study, and the possibility of other co-infections, demonstrate that the development of a multiplex PCR assay for use in the field and in the laboratory would be a great benefit for the differential diagnosis of PPR-like disease cases in situations where diseases with similar clinical signs are prevalent, such as bluetongue, sheep and goat pox, CCPP and FMD. Such assays need to be operationalised in PPR-endemic areas to support improved disease surveillance and targeted interventions to mitigate disease impact and to eliminate PPRV.

Phylogenetic analysis of partial N gene sequences generated in this study further confirmed the circulation of lineage III virus in northern Tanzania. These partial sequences were most closely related to a 2013 isolate from Ngorongoro, Tanzania (Figure 7). The PPR epidemiological situation in Tanzania appears to be complex, as three (II, III and IV) out of four lineages have been detected since PPRV was first confirmed in 2008 [22,40,64]. Variation in the virulence of different strains of PPRV has been demonstrated under experimental conditions [65]. However, based on partial genome sequencing, the virus strains detected in this study were almost identical, and therefore the variation in observed clinical signs between the four flocks from which sequences were obtained (Table 1) was most likely due to host or environmental factors or co-infections rather than virus genotype. The circulation of multiple lineages of PPRV in Tanzania reflects multiple introductions, most likely through transboundary movements of infected small ruminants for trade or migration from neighbouring countries where PPRV is also considered to be endemic and multiple lineages have been detected—Kenya, Uganda, Rwanda, Burundi and the Democratic Republic of the Congo [66,67,68,69,70]. However, the live attenuated PPRV vaccine (Nigeria/75/1) is reported to provide protection against all the lineages [71].

## 5. Conclusions

The wide variation in the clinical presentation of PPR disease in this ecosystem makes the diagnosis of PPR challenging for livestock keepers, animal health workers and veterinarians alike. Awareness of the variability of PPR clinical disease and local disease names should be included in communication materials and training courses for PPR surveillance to improve PPR reporting and diagnosis. Field veterinarians should have greater access to pen-side tests including multiplex assays for PPRV differential diagnosis for improved information on PPRV occurrence to allow targeting of PPRV eradication efforts. The variations in clinical presentation between flocks that were observed in these investigations were unlikely to be due to differences in virus genotype, but were more likely to be due to host or environmental factors or co-infections. Despite the presence of large herds of wild artiodactyls in proximity to domestic flocks, no reports or signs of disease syndromes similar to PPR were noted.

## Figures and Tables

**Table 1:** Summary of the characteristics of confirmed peste des petits ruminants (PPR) outbreaks and diagnostic test results in Ngorongoro District, June–July 2015.

Ward	Flock No.	Local Disease Name (Maa Language)	Species/Age Groups Affected	Clinical Signs										Flock Mortality (%)	Flock Morbidity ^†^ (%)	Diagnostic Test Results Number Positive (Number Tested)					Partial N Gene Sequence
				Pyrexia	Lacrimation	Nasal Discharge	Sneezing	Coughing	Dyspnoea	Peri-Oral Lesions	Mouth Lesions	Salivation	Diarrhoea			PPRV-RDT	PPRV RT-qPCR	PPRV cELISA	BTV RT-qPCR	Capripox qPCR	
Olorien-Magaiduru	1	*olodua*	sheep and goats, all ages	X	X	X	X	X	-	X	X	-	X	2.2	66.6	2 (3)	4 (7)	1 (4)	1 (4)	0 (4)	TANZANIAGoat3/2015, TANZANIAGoat4/2015
	3	*olkipiei*	sheep and goats, all ages	X	X	X	-	X	-	-	X	-	X	1.5	60.0	2 (3)	3 (3)	2 (2)	--	--	TANZANIAGoat10/2015, TANZANIAGoat11/2015
	4	*olodua*	goats	-	-	X	-	-	-	X	-	-	X	3.0	5.5	1 (1)	--	--	--	--	
	9	*oloirobi*	sheep and goats (more sheep affected than goats)	X	-	X	X	X	-	X	X	X	X	9.1	15.9	0 (3)	2 (6)	1 (3)	1 (1)	0 (1)	
Soitsambu and Ololosokwan	5	*olodua*	adult sheep	-	-	X	-	-	X	X	X	X	-	0.2	4.3	1 (2)	2 (2)	1 (2)	--	--	TANZANIASheep14/2015
	6	*olodua* or *olkipiei*	sheep and goats, all ages	X	X	X	-	-	X	X	-	X	X	2.0	14.0	1 (1)	2 (2)	1 (1)	0 (1)	0 (1)	TANZANIASheep15/2015
	17	none	sheep and goats	-	X	X	-	-	-	X	X	X	-	NA	NA	--	2 (3)	1 (2)	--	--	
Olbalbal	19	*olkipiei*	Goats only, mainly young	X	-	X	-	-	X	X	X	-	X	25.0	31.3	0 (2)	1 (5)	0 (2)	0 (1)	0 (1)	
Endulen	26	NA		-	-	X	-	-	-	-	X	-	X	NA	NA	--	1 (3)	1 (3)	--	--	
Kakesio	29	*enkorotik*		-	X	X	-	-	-	-	X	-	X	4.0	4.6	1 (2)	1 (2)	3 (5)	--	--	
Total number positive (total number tested)																8 (17)	18 (33)	11 (24)	2 (7)	0 (7)	

^†^ Includes sick and dead. Abbreviations: NA = not available, PPRV = peste des petits ruminants virus, BTV = bluetongue virus, RDT = rapid detection test, RT-qPCR = real-time reverse transcription-polymerase chain reaction, qPCR = real-time polymerase chain reaction, and cELISA = competitive enzyme-linked immunosorbent assay. X = present, - = not present; -- = not tested. Maa language disease names: *enkorotik* means diarrhoea, *olkipiei* means “lung”, a term used for disease of the lungs, *olodua* means “bile” or “gall bladder”, a term used for rinderpest in cattle, and *oloirobi* means “fever”.

**Table 2:** Descriptive, univariable and multivariable analysis of the number of sheep and goats with clinical signs by species and age group in flocks 1 and 3 (*n* = 831).

Variable	Category	Number of Animals with Clinical Signs (%)	Total Number of Animals	Univariable Analysis χ^2^ Test *p* Value	Adjusted Odds Ratio (95% Confidence Interval)	Multivariable Logistic Regression Wald Test *p* Value
Flock	1	510 (66.58)	766	0.282	1.01 (0.77, 1.33)	0.95
	3	39 (60.00)	65			
Species	Goat	211 (60.29)	350	0.003	1.60 (1.17, 2.17)	0.003
	Sheep	338 (70.27)	481			
Age	>12 months	273 (61.35)	445	0.002	1.61 (1.20, 2.16)	0.001
	<12 months	276 (71.50)	386			

**Table 3:** Comparison of peste des petits ruminants virus (PPRV) rapid detection test (RDT) and PPRV real-time reverse transcription polymerase chain reaction (RT-qPCR) results.

Diagnostic Test		PPRV RT-qPCR		Total
	Test Result	Positive	Negative	
PPRV rapid detection test (RDT)	Positive	6	0	6
	Negative	5	4	9
	Total	11	4	15

## Supplementary Materials

The following are available online at https://www.mdpi.com/1999-4915/12/4/389/s1, File S1: Outbreak investigation form; Table S1: Summary of investigations of PPR-like disease reports in Ngorongoro District, June–July 2015; Figure S1: A suspected outbreak of CCPP.
