# Supplementary material for: Characterisation of Peste Des Petits Ruminants Disease in Pastoralist Flocks in Ngorongoro District of Northern Tanzania and Bluetongue Virus Co-Infection
Source: Viruses. 2020 Mar 31;12(4):389. doi: 10.3390/v12040389 (PMC7232183; doi:10.3390/v12040389)
Supplement: Supplementary file 1 [file viruses-12-00389-s001.zip › SI information/Figure S1.docx]

Figure S1 A suspected outbreak of CCPP

Photographs from Flock 2 in Soitsambu – a suspected outbreak of contagious caprine pleuropneumonia (CCPP)


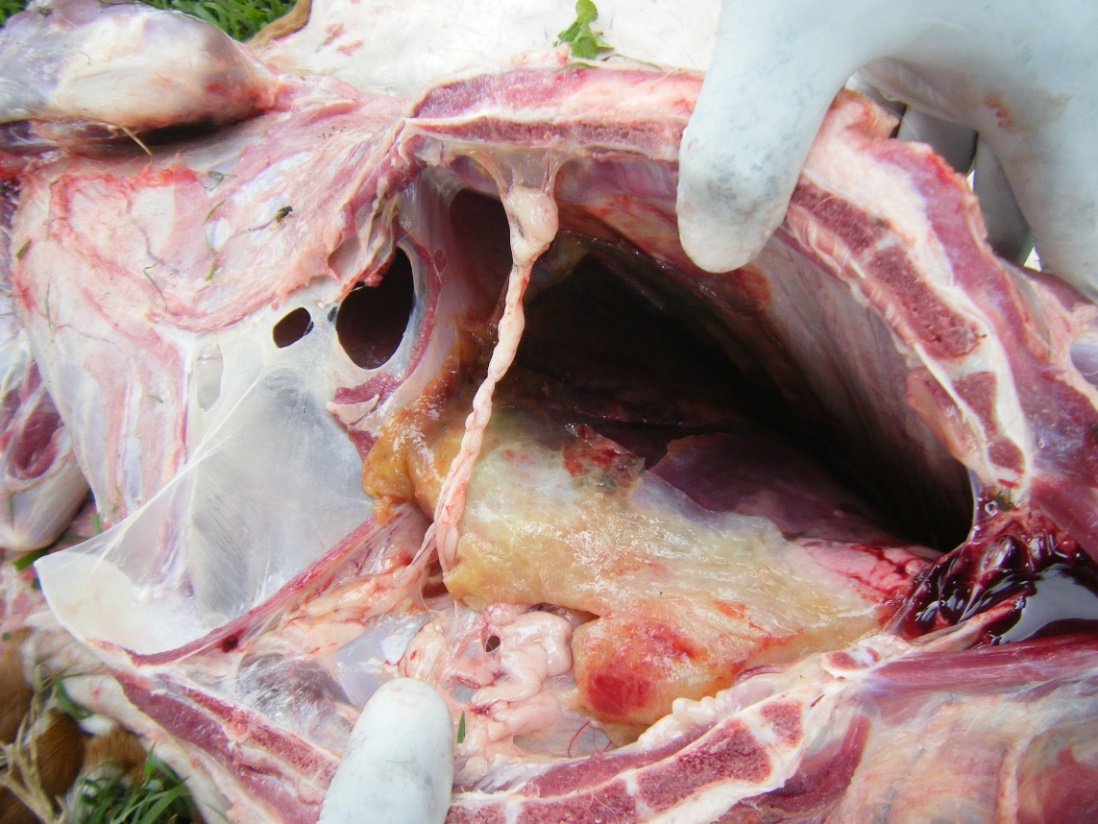


1. Post mortem examination of a three-year old goat with nasal discharge and dyspnoea. The thoracic cavity has been opened (cranial end to the right of the picture) showing a large yellow fibrin clot in the ventral thorax.


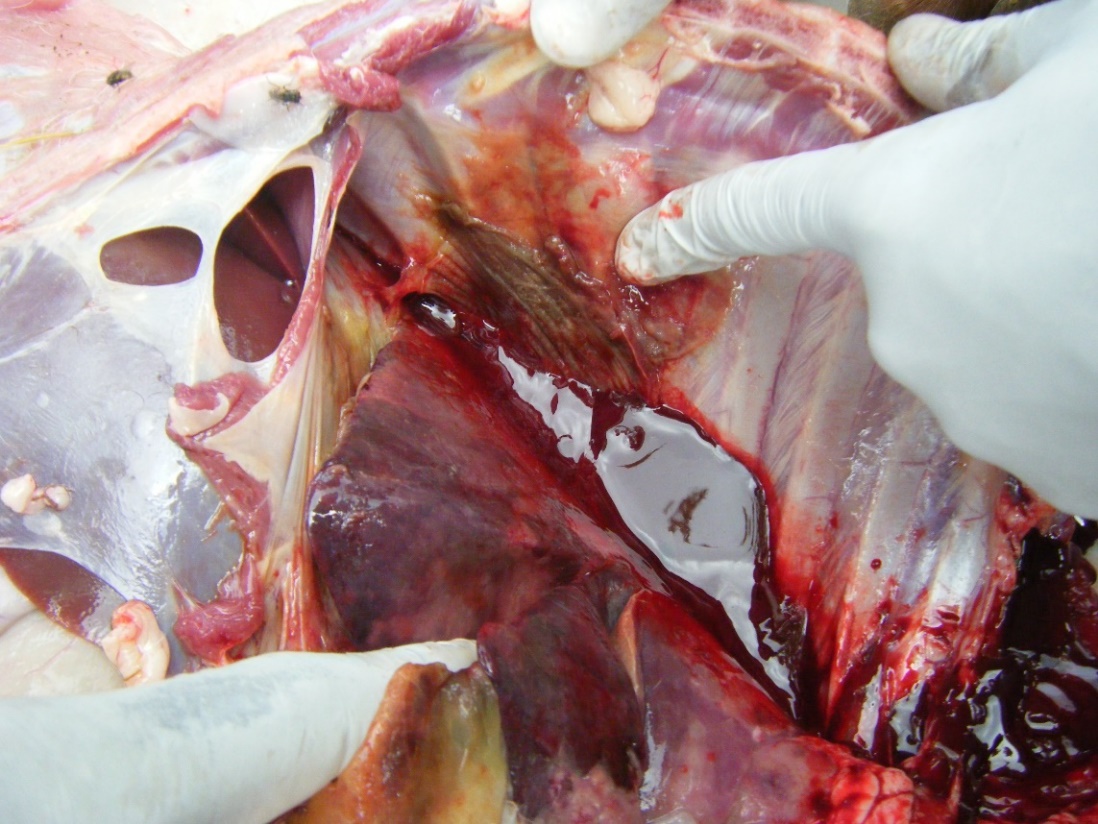


1. The same goat showing adhesions to the wall of the thorax and almost complete hepatisation of the lung.
