# Supplementary material for: Characterisation of Peste Des Petits Ruminants Disease in Pastoralist Flocks in Ngorongoro District of Northern Tanzania and Bluetongue Virus Co-Infection
Source: Viruses. 2020 Mar 31;12(4):389. doi: 10.3390/v12040389 (PMC7232183; doi:10.3390/v12040389)
Supplement: Supplementary file 1 [file viruses-12-00389-s001.zip › SI information/S1 Outbreak investigation form.docx]

**File S1 Outbreak Investigation Form**

| Name of owner |  | Date of investigation |  |  |
| --- | --- | --- | --- | --- |
| Village |  | GPS Coordinates |  |  |
| Ward |  | Number of sheep (approx.) |  |  |
| District |  | Number of goats (approx.) |  |  |

1. *Date of onset of first case:*
2. *Animals affected*

|  | Goats | Sheep | Remarks (age groups, etc. |
| --- | --- | --- | --- |
| No. sick |  |  |  |
| No. dead |  |  |  |

1. *Clinical signs*

|  | Goats | Sheep |
| --- | --- | --- |
| Clinical signs |  |  |
| Abortions? |  |  |
| Effect on milk production? |  |  |
| Effect on body condition/growth? |  |  |
| Post mortem signs |  |  |

1. *What local name does the owner give for this disease?*
2. *Has he or she seen this disease before in their own herd/flock? When?*
3. *Are any other herds/flocks in the area also affected? (number of herds/flocks, location, date of onset, contact with this flock)*
4. *What treatment or control measures have been applied? What effect was observed?*
5. *Has the herd/flock ever been vaccinated against PPR? (give the dates) other diseases?*
6. *What other disease problems has the owner seen in the herd/flock recently or in the past?*
7. *Contact with other flocks (water points, grazing, etc.)*
8. *Has the flock migrated recently?*
9. *Did any sheep or goats join the herd/flock in the last one month? From where? And why?*
10. *Has the herd/flock been in contact with any wild animals? (give names of wild animals, location, type of contact)*
11. *Have they ever seen sick wild animals? (species, clinical signs, deaths)*

*Other remarks*

**Clinical examination of sick animals**

| No. | Species | | Sex | | | Dentition (pairs perm incisors) | | | | | | | Age | Temp  ⁰C | Ocular discharge, other | Nasal discharge | Oro-nasal lesions | Respiratory signs, rate | Faeces | Remarks |
| --- | --- | --- | --- | --- | --- | --- | --- | --- | --- | --- | --- | --- | --- | --- | --- | --- | --- | --- | --- | --- |
|  | G | Sh | M | F | Mc | 0 | 1 | 2 | 3 | 4 | 4* | 4** |  |  |  |  |  |  |  |  |
| 1 |  |  |  |  |  |  |  |  |  |  |  |  |  |  |  |  |  |  |  |  |
| 2 |  |  |  |  |  |  |  |  |  |  |  |  |  |  |  |  |  |  |  |  |
| 3 |  |  |  |  |  |  |  |  |  |  |  |  |  |  |  |  |  |  |  |  |
| 4 |  |  |  |  |  |  |  |  |  |  |  |  |  |  |  |  |  |  |  |  |
| 5 |  |  |  |  |  |  |  |  |  |  |  |  |  |  |  |  |  |  |  |  |
| 6 |  |  |  |  |  |  |  |  |  |  |  |  |  |  |  |  |  |  |  |  |
| 7 |  |  |  |  |  |  |  |  |  |  |  |  |  |  |  |  |  |  |  |  |
| 8 |  |  |  |  |  |  |  |  |  |  |  |  |  |  |  |  |  |  |  |  |
| 9 |  |  |  |  |  |  |  |  |  |  |  |  |  |  |  |  |  |  |  |  |
| 10 |  |  |  |  |  |  |  |  |  |  |  |  |  |  |  |  |  |  |  |  |
| 11 |  |  |  |  |  |  |  |  |  |  |  |  |  |  |  |  |  |  |  |  |
| 12 |  |  |  |  |  |  |  |  |  |  |  |  |  |  |  |  |  |  |  |  |

Notes: 1. Ocular discharge/other: watery, mucoid, purulent, redness, congestion, opacity. 2. Nasal discharge/other: watery, mucoid, purulent, lesions. 3. Oral lesions/other: congestion, lesion type, limited/widespread, salivation. 4. Respiratory signs/rate: tachypnoea (normal goat 10-30/min, sheep 15-40/min), cough, dyspnoea. 5. Faeces: firm/soft/runny, haemorrhage, mucus. 6. Remarks: general condition, activity, depression, appetite, able to stand
