# Supplementary material for: Characterisation of Peste Des Petits Ruminants Disease in Pastoralist Flocks in Ngorongoro District of Northern Tanzania and Bluetongue Virus Co-Infection
Source: Viruses. 2020 Mar 31;12(4):389. doi: 10.3390/v12040389 (PMC7232183; doi:10.3390/v12040389)
Supplement: Supplementary file 1 [file viruses-12-00389-s001.zip › SI information/Table S1.docx]

#### Table SI. Summary of Investigations of peste des petits ruminants (PPR)-like disease reports in Ngorongoro District, June-July 2015

Confirmed PPR virus (PPRV) infected flocks are shaded in grey. A flock was considered to have confirmed PPRV infection if one or more animals was positive by PPRV-rapid detection test (PPRV-RDT) and/or positive by PPRV real-time reverse transcription-polymerase chain reaction (RT-qPCR). A flock was considered to have confirmed bluetongue virus (BTV) infection if one or more animals was positive by BTV RT-qPCR.

| **Flock**  **No.** | **Disease name in Maa language** | **Main clinical signs observed** | **PPR vaccination** | **Number died (%)** | **Number sick† (%)** | **Flock size‡ (% goats)** | **Diagnostic test results**  **Number positive (number tested)** | | | | | **Diagnosis** |
| --- | --- | --- | --- | --- | --- | --- | --- | --- | --- | --- | --- | --- |
|  |  |  |  |  |  |  | ***PPRV-RDT*** | **PPRV RT-qPCR** | **PPRV**  **cELISA** | **BTV RT-qPCR** | ***Capripox qPCR*** |  |
| **Olorien-Magaiduru Ward** | | | | | | | | | | | | |
| 1 | *olodua* | Pyrexia, nasal discharge, sneezing, lacrimation, coughing, diarrhoea, peri-oral skin lesions, oral lesions. 1 abortion. Sheep & goats, all ages. | None | 17 (2.2) | 517 (66.6) | 776 (37.5) | 2 (3) | 4 (7) | 1 (4) | 1 (4) | 0 (4) | **PPRV confirmed**  Partial N-gene sequences; TANZANIAGoat3/2015, TANZANIAGoat4/2015  **BTV confirmed** |
| 3 | *olkipiei* | Pyrexia, mucoid nasal discharge, lacrimation, coughing, oral lesions, sub-mandibular oedema, diarrhoea. 2 abortions. Sheep and goats, all ages. | 2013 | 1 (1.5) | 39 (60.0) | 65 (3.1) | 2 (3) | 3 (3) | 2 (2) | - | - | **PPRV confirmed**  Partial N-gene sequences; TANZANIAGoat10/2015, TANZANIAGoat11/2015 |
| 4 | *olodua* | Diarrhoea, nasal discharge, peri-oral skin lesions, weight loss. | 2012 | 6 (3.0) | 11 (5.5) | 200 (95.0) | 1 (1) | - | - | - | - | **PPRV confirmed** |
| 7 | *oltikana* | Pyrexia, lacrimation, nasal discharge in sheep. | NA | 2 (NA) | 5 (NA) | NA | 0 (1) | - | - | - | - | ? |
| 8 | *olomoroj* | Pyrexia, lacrimation, nasal discharge, skin nodules over face and body, diarrhoea – young goats only. | NA | 0 | 6 (4.0) | 150 (50.0) | - | - | - | - | - | Goat pox (clinical) |
| 9 | *oloirobi* | Nasal discharge, sneezing, coughing, sheep & goats. Some sheep had pyrexia, frothy salivation, swollen ulcerated muzzle, mouth erosions, diarrhoea. | 2012  1 month ago¶ | 60 (9.1) | 105 (15.9) | 660 (37.9) | 0 (3) | 2 (6) | 1 (3) | 1 (1) | 0 (1) | **PPRV confirmed**  **BTV confirmed** |
| 10 | *olodua* | Coughing, sneezing, nasal discharge, diarrhoea in sheep & goats. One with mouth lesions. | 2012  1 month ago¶ | 25 (4.3) | 175 (30.4) | 575 (41.7) | - | - | - | - | - | Suspected PPRV |
| 11 | None | Pyrexia, nasal discharge, diarrhoea. Affected animals born/brought in since vaccination 6 months ago. | Jan 2015 | 8 (4.5) | 13 (7.3) | 178 (50.0) | - | - | - | - | - | Suspected PPRV |
| **Soitsambu and Ololosokwan Ward** | | | | | | | | | | | | |
| 2 | *olkipiei* mixed with *olodua* | Pyrexia, nasal discharge, diarrhoea, dyspnoea. PME: pleuropneumonia, fibrinous clots, pleural adhesions. One abortion. Goats only. | 2013  1 month ago¶ | 64 (26.2) | 78 (32.0) | 244 (66.7) | 0 (1) | 0 (1) | 3 (3) | 1 (1) | 0 (1) | Suspected CCPP  **BTV confirmed** |
| 5 | *olodua* | Nasal discharge, profuse frothy salivation, sores in and around mouth. Affecting adult sheep. | None | 1 (2.1) | 20 (4.3) | 470 (21.3) | 1 (2) | 2 (2) | 1 (2) | - | - | **PPRV confirmed**  Partial N-gene sequence; TANZANIASheep14/2015 |
| 6 | *olodua* or *olkipiei* | Pyrexia, nasal discharge, lacrimation, salivation, diarrhoea, sores around mouth, dyspnoea. Sheep and goats, all ages. | 2012 | 10 (2.0) | 70 (14.0) | 500 (NA) | 1 (1) | 2 (2) | 1 (1) | 0 (1) | 0 (1) | **PPRV confirmed**  Partial N-gene sequence; TANZANIASheep15/2015 |
| 16 | *oloikirobi* | Pyrexia, nasal discharge, sores on nose, erosions in mouth, diarrhoea. Sheep only. | 2014 | 1 (0.4) | 30 (12.0) | 250 (12.0) | 0 (1) | 0 (3) | 0 (3) | - | - | Suspected PPR, differentials FMD, BTV |
| 17 | None | Nasal discharge, frothy salivation, erosions on dental pad, lacrimation, peri-oral nodules. Two abortions. | 2012 | 4 (NA) | 2 (NA) | NA | - | 2 (3) | 1 (2) | **-** | **-** | **PPRV confirmed** |
| 18 | *olodua* or *oloirobi* | Lacrimation, mucoid nasal discharge, white coating on dental pad & tongue, green watery diarrhoea. | 2 month ago¶ | 10 (NA) | 100 (NA) | NA | - | 0 (1) | 1 (1) | - | - | Suspected PPR, differentials FMD, BTV |
| **Engoserosambu Ward** | | | | | | | | | | | | |
| 12 | none | Nasal discharge, sneezing, coughing, diarrhoea. | 2012 | NA | NA | 700 (30.0) | - | - | - | - | - | ? |
| 13 | none | Immature sheep with diarrhoea | 2012 | 13 (NA) | 20 (NA) | NA | - | - | - | - | - | Suspected parasitic gastro-enteritis |
| 14 | none | Pyrexia, recumbent, stiff, crying. PM: green turbid joint & pericardial fluid, congested meninges, oedematous mesenteric lymph nodes. | NA | 2 (NA) | 3 (NA) | NA | - | - | - | - | - | Bacterial meningitis, septicaemia. |
| 15 | none | Pyrexia, diarrhoea, nasal discharge, sub-mandibular oedema, weight loss. | 2012 | 10 (NA) | 20 (NA) | NA | - | - | - | - | - | Suspected parasitic gastro-enteritis |
| **Olbalbal Ward** | | | | | | | | | | | | |
| 19 | *olkipiei* | Pyrexia, eye and nose discharge, dyspnoea, diarrhoea, abdominal distension, peri-oral and oral lesions in young. 30 goat abortions. | 2013 | 200 (25.0) | 250 (31.3) | 800 (50.0) | 0 (2) | 1 (5) | 0 (2) | 0 (1) | 0 (1) | **PPRV confirmed** |
| 20 | *olkipiei* | Coughing, nasal discharge, dyspnoea, diarrhoea. PM: lung oedema and hepatisation, enlarged bronchial lymph nodes. | 2010 &  1 month ago | 180 (45.0) | NA | 400 (NA) | - | 0 (1) | 1 (1) | - | - | Suspected CCPP. |
| 21 | *olkipiei* | Pyrexia, coughing, lacrimation, nasal discharge, dyspnoea, diarrhoea. | NA | 17 (10.0) | NA | 170 (NA) | - | - | - | - | - | Suspected CCPP. |
| **Endulen Ward** | | | | | | | | | | | | |
| 31 | None | No current cases. | NA | 2 (25.0) | 2 (25.0) | 8 (100.0) | - | - | 3 (3) | - | - | ? |
| 32 | None | Diarrhoea. | NA | 1 (3.3) | 2 (6.7) | 30 (100.0) | - | - | - | - | - | ? |
| 33 | None | Nasal discharge, lacrimation. | 2013 | 7 (4.7) | 13 (8.7) | 150 (50.0) | - | - | 1 (3) | - | - | ? |
| 22 | *enkorotik* | Diarrhoea, slight lacrimation. | 2012 | 20 (20.0) | 21 (21.0) | 100 (50.0) | - | - | 0 (2) | - | - | ? |
| 23 | *enkorotik* | Nasal discharge, lacrimation, diarrhoea, coughing. | 2012 | 15 (6.0) | NA | 250 (50.0) | - | 0 (3) | 0 (3) | - | - | ? |
| 24 | NA | Nasal discharge, diarrhoea. | None | 10 (3.3) | 14 (4.7) | 300 (0.0) | - | - | 0 (3) | - | - | ? |
| 25 | NA | Pyrexia, nasal discharge, lacrimation, diarrhoea. | None | 4 (2.0) | 9 (4.5) | 200 (50.0) | - | 0 (3) | 0 (3) | - | - | Suspected PPR |
| 26 | NA | Nasal discharge, diarrhoea, mouth lesions. | None | NA | NA | 300 (50.0) | - | 1 (3) | 1 (3) | **-** | **-** | **PPRV confirmed** |
| **Kakesio Ward** | | | | | | | | | | | | |
| 27 | *enkorotik* | Nasal discharge, coughing, diarrhoea. | 2010 | 3 (2.3) | 16 (12.3) | 130 (50.0) | - | - | 1 (2) | - | - | ? |
| 28 | *enkorotik, harisha* | Lacrimation, nasal discharge, diarrhoea. | NA | 5 (1.7) | 7 (2.3) | 300 (83.0) | - | - | - | - | - | ? |
| 29 | *enkorotik* | Diarrhoea, lacrimation, nasal discharge, mouth lesions. | None | 20 (4.0) | 23 (4.6) | 500 (50.0) | 1 (2) | 1 (2) | 3 (5) | **-** | **-** | **PPRV confirmed** |
| 30 | NA | A few recovering animals; sub-mandibular oedema, swelling around eyes, healing mouth lesions. | 2012 | 300 (46.1) | 303 (46.6) | 650 (10.0) | - | - | 2 (3) | - | - | ? |
| Total number positive (total number tested) | | | | | | | 8 (21) | 18 (45) | 23 (54) | 3 (8) | 0 (8) |  |

† includes sick and dead

‡ includes dead

¶ vaccination carried out privately by livestock keeper of unknown efficacy

Abbreviations: NA = not available, “-“ = not tested, FMD = foot-and-mouth disease, CCPP = contagious caprine pleuropneumonia, qPCR = real-time polymerase chain reaction, cELISA competitive enzyme-linked immunosorbent assay.

Maa language disease names:

*enkorotik* means diarrhoea, and *harisha* means diarrhoea in Swahili

*olkipiei* means “lung”, a term used for disease of the lungs

*olodua* means “bile” or “gall bladder”, a term used for rinderpest in cattle

*olomoroj* means “nodules”

*oloirobi, oloikirobi* mean “fever”

*oltikana* is a term used for East Coast fever, a disease of cattle
